# What every paediatrician needs to know about mechanical ventilation

**DOI:** 10.1007/s00431-024-05793-z

**Published:** 2024-09-30

**Authors:** Jeroen ter Horst, Peter C. Rimensberger, Martin C. J. Kneyber

**Affiliations:** 1 Division of Paediatric Critical Care Medicine, Department of Paediatrics, Beatrix Children’s Hospital, University Medical Center Groningen, University of Groningen, Huispost CA62, P.O. Box 30.001, 9700 RB Groningen, the Netherlands; 2 Division of Neonatology and Paediatric Intensive Care, University of Geneva, Geneva, Switzerland; 3 Critical Care, Anaesthesiology, Peri-Operative & Emergency Medicine (CAPE), University of Groningen, Groningen, the Netherlands

**Keywords:** Paediatrician, Mechanical ventilation, Ventilation-induced lung injury

## Abstract

Invasive mechanical ventilation (MV) is one of the most practiced interventions in the intensive care unit (ICU) and is unmistakably lifesaving for children with acute respiratory failure (ARF). However, if delivered inappropriately (i.e. ignoring the respiratory system mechanics and not targeted to the need of the individual patient at a specific time point in the disease trajectory), the side effects will outweigh the benefits. Decades of experimental and clinical investigations have resulted in a better understanding of three important detrimental effects of MV. These are ventilation-induced lung injury (VILI), patient self-inflicted lung injury (P-SILI), and ventilation-induced diaphragmatic injury (VIDD). VILI, P-SILI, and VIDD have in common that they occur when there is either too much or too little ventilatory assistance.

*Conclusion*: The purpose of this review is to give the paediatrician an overview of the challenges to prevent these detrimental effects and titrate MV to the individual patient needs.

## Introduction

Invasive mechanical ventilation (MV) is one of the most practiced interventions in the intensive care unit (ICU) and is unmistakably lifesaving for children with acute respiratory failure (ARF). The advent of its use marked the start of modern-day ICUs when Bjorn Ibsen, an anaesthesiologist in Copenhagen (Denmark), treated patients suffering from polio-induced ARF with the delivery of positive pressure ventilation through a tracheostomy. In the subsequent decades, many technical aspects of ventilators significantly improved but at the same time more and more was learned about the side-effects of MV. Basically, if MV is delivered inappropriately (i.e. ignoring the respiratory system mechanics and not targeted to the need of the individual patient at a specific time point in the disease trajectory), the side effects will outweigh the benefits. Decades of experimental and clinical investigations have resulted in a better understanding of three important detrimental effects of MV. These are ventilation-induced lung injury (VILI), patient self-inflicted lung injury (P-SILI), and ventilation-induced diaphragmatic injury (VIDD). The purpose of this review is to give an overview of the practice and challenges of MV that need to be considered when setting and titrating ventilator settings to meet the individual needs of a patient.

## What is MV?

MV entails the delivery of positive pressure through an oral or nasal endotracheal tube (ETT) or a tracheostomy. There are three different forms of MV (i.e. continuous mandatory ventilation (CMV), continuous intermittent ventilation (CIV), and continuous spontaneous ventilation (CSV)) with two different control variables (i.e. volume-controlled (VC) or pressure-controlled (PC)). As such, there are five different modes of ventilation (i.e. VC-CMV, VC-IMV, PC-CMV, PC-IMV, and PC-CSV). The difference between CMV and CSV is that with the first mode of ventilation, all breaths are delivered by the ventilator and that there is no possibility for spontaneous breathing, whereas in CSV mode, all breaths are spontaneous and thus generated by the patient. IMV is a mode of ventilation where intermittent mandatory breaths are delivered at clinician-defined intervals, and between these mandatory breaths, the patient can breathe spontaneously without receiving any support. Synchronized Intermittent Mandatory Ventilation (SIMV) is an IMV mode where the ventilator delivers a preset number of mandatory breaths per minute while attempting to synchronize the delivery of these mandatory breaths with the spontaneous efforts of the patient. Patient breaths above the set ventilator rate can be supported by an additional pressure support or not. This mode is often used as a first ventilator mode in the paediatric intensive care unit (PICU), although it was initially developed for ventilator weaning, However, this ventilator mode is prone for asynchrony (i.e. a mismatch between patient demand and ventilator delivery) with its various consequences (e.g. patient discomfort).

## Basic physiology to understand mechanical ventilation

The equation of motion describes the pressure that the ventilator must generate to overcome the elastic and resistive load in a passive (i.e. not breathing spontaneously) mechanically ventilated patient:

1 $$\text{Pventilator }= (\text{resistance }\times \text{ flow}) + (\text{elastance }\times \text{ change in volume}) +\text{ pressure before initiation of breath}$$

Elastance is the reciprocal of compliance (which is calculated by ∆volume divided by ∆pressure) and describes the elastic recoil pressures of the respiratory system. In an actively breathing patient, the muscular pressure (Pmus) needs to be added to the Pventilator (Ptotal = Pventilator + Pmus).

Pventilator (or Ptotal) is necessary to inflate the lungs, whereas the pressure at the end of expiration (i.e. positive end-expiratory pressure (PEEP)) is set to maintain alveolar patency (i.e. prevent alveolar collapse), thereby maintaining end-expiratory lung volume (EELV).

From the equation of motion, it can be appreciated that the pressure measured at the airway opening does not reflect the pressure at alveolar level; this pressure can be estimated during zero-flow states (to eliminate resistive effects) and is known as the plateau pressure (Pplat). The pressure that distends the alveoli is called the end-inspiratory transpulmonary pressure (Plung) and is calculated by the difference between Pplat and the pleural pressure (Ppl). Ppl cannot be measured directly but can be approximated by measuring the oesophageal pressure (Poes). Plung reflects lung stress, i.e. the retracting force experienced by the stretched lung unit area; lung strain is reflected by the change in Vt over EELV, i.e. the deformation (size and shape) of the lung structure during a tidal breath. Lung stress and strain are intimately linked through the specific lung elastance which is similar for children and adults [1, 2]. Airway driving pressure (i.e. the difference between airway pressure at zero-flow conditions and positive end-expiratory pressure) can detect lung overstress with an acceptable accuracy [3].

## Ventilation-induced lung injury

VILI is an overarching term indicating the structural and physiological lung changes caused by MV. In the 1930s, it was observed in experimental studies that MV caused alveolar rupture that lead to gas escaping along the pulmonary vascular sheaths causing pneumomediastinum, subcutaneous emphysema, and pneumothorax [4]. About 50 years later, the term “barotrauma” to describe VILI was introduced when it was observed that ventilating with high peak inspiratory pressures resulted in increased microvascular permeability and lung injury in among others the seminal study by Webb and Tierney [5–7]. Over the next years, Dreyfuss and co-workers identified that ventilating with supraphysiologic tidal volume (Vt) resulted in VILI thereby giving birth to the term “volutrauma”. Tremblay and co-workers demonstrated that atelectasis caused lung inflammation due to increased pressure at the interface of open and closed alveoli, which became known as “atelectrauma” [8, 9]. It became also apparent that injurious forms of ventilation that resulted in overdistension or atelectasis could lead to a release of inflammatory mediators in the lung (“biotrauma”) and that these mediators could spill over in the systemic circulation [8]. Many of the mechanisms underlying VILI come together in lung stress and strain.

Mechanical power (MP) has been proposed as unifying determinants of VILI [10]. It is an estimate of the mechanical energy per minute being applied to the respiratory system. The attractiveness of the concept of MP lies in the fact that it integrates the individual components of lung stress and strain such as volume and pressures with respiratory rate and flow, both of which may also contribute to VILI. In adults with acute respiratory distress syndrome (ARDS), higher mechanical power has been associated with higher mortality (4–6). In children, MP and surrogates for MP normalised to bodyweight to overcome the age-dependency of specific variables to calculate MP (coined mechanical energy) has also been linked to longer total ventilation time and lower ventilator-free days [11, 12].

In adults, the importance of volume setting during MV was underscored the National Heart, Lung and Blood Institute ARDS Network trial reported lower mortality rates in critically ill adults with ARDS randomized to low Vt ventilation (i.e. 6 mL/kg ideal bodyweight [IBW]) and plateau pressures (Pplat) less than 30 cmH_2_O compared to 12 mL/kg IBW [13] and Pplat < 50 cmH_2_O [13]. Paediatric pre-clinical models also confirmed deleterious effects of injurious MV [14]. In clinical studies, a pro-inflammatory response was observed in one small study of 12 infants without pre-existing lung injury elective ventilated for 2 h with a Vt of 10 mL/kg, thereby suggesting that the paediatric lung may also be susceptible to MV-induced stretch even in the absence of lung injury [15]. One group of investigators observed lower mortality among children ventilated with Vt ~ 8 mL/kg actual bodyweight compared with ~ 10 mL/kg in a before-after retrospective study [16]. Others have reported that failure to reduce Vt with increasing lung severity was associated with increased mortality in PARDS patients [17]. With inspiratory pressures and PEEP, a direct relationship between peak inspiratory pressure (PIP) and mortality has been observed in retrospective and observational studies of children with (severe) lung injury [18–21]. It is common that low levels of PEEP are used and inherently higher FiO2_2_ are accepted [22]. However, such practices are not free from harm as higher increased mortality has been reported among PARDS patients [23, 24]. Driving pressure > 15 cmH_2_O and mechanical power or mechanical energy (which is the amount of energy delivered per breath) has been linked with fewer ventilator-free days at day 28 (VFD-28), which is a composite endpoint of mortality and duration of MV, and long duration of ventilation [11, 17, 25].

## Patient self-inflicted lung injury

The beneficial effects of having mechanically ventilated patients breathe spontaneously (i.e. triggering the ventilator or being in a continuous spontaneous ventilation mode) include preferential distribution of the tidal volume towards the dorsal, well–perfused regions of the lung, thereby reducing shunt fraction and reducing inflammation [26–31]. However, it became clear that strenuous, sustained spontaneous breathing especially in the presence of severe lung injury may also contribute to lung injury, a phenomenon known as patient self-inflicted lung injury (P-SILI). [32–35] Experimental work showed under these circumstances spontaneous breathing promoted global and regional lung stress and strain and subsequent lung inflammation [32, 36, 37]. Thus, VILI and P-SILI share pathophysiological mechanisms, albeit that vigorous spontaneous breathing will lead to disproportionally more vascular than epithelial injury as seen during VILI, potentially related to negative pressure swings, and increased pulmonary blood flow [32, 36, 38]. Pendelluft involves the movement of air from non-dependent lung regions (with less injury and more compliance) to dependent lung regions (with more injury and less compliance) within the same breath [32]. This internal lung movement can result in overdistension and further injury of the dependent lung regions.

There is indirect evidence for P-SILI in children. A secondary analysis from the Randomized Evaluation of Sedation Titration for Respiratory Failure (RESTORE) trial reported a relationship between duration of non-invasive ventilation (NIV) use before intubation and worse outcomes in children with acute respiratory failure [39]. Another group of investigators reported similar findings through a database analysis adjusting for disease severity of data from over 5.000 children [40]. By design, non-invasive respiratory support relies on spontaneous breathing. It may therefore be surmised at least in a group of patients from these studies P-SILI may have occurred explaining adverse outcomes.

## Ventilation-induced diaphragmatic dysfunction

Following early observations on alterations in diaphragm structure during MV in neonates and adults, it is becoming clear that ventilation-induced diaphragmatic dysfunction (VIDD) can also develop during the course of MV, although it must be appreciated that also other respiratory muscles can be affected [41–43]. Diaphragm atrophy and injury (also known as “myotrauma”) may occur via several, of which the most well-established mechanism is ventilatory overassistance. This leads to excessive diaphragmatic unloading because the patient inspiratory efforts are strongly reduced, resulting in atrophy. On the opposite, excessive diaphragm loading due to insufficient ventilator assistance can induce acute muscle inflammation and injury, resulting in diaphragm thickening [44]. Changes in diaphragm thickness (T_di_) correlate with diaphragm contractile activity, duration of MV and patient outcome [45, 46]. Other mechanisms of myotrauma include eccentric contraction of the diaphragm during expiration, non-synchronised bilevel ventilation modes and patient–ventilator dyssynchrony (in particular reverse triggering, premature cycling, and ineffective triggering). Also, maintaining high levels of PEEP may cause diaphragm longitudinal atrophy.

There is increasing data reporting diaphragm myotrauma in ventilated children. Several investigators reported a significant decrease diaphragm thickness and diaphragm thickening fraction over time [47–51]. Changes in diaphragm thickness occurred already after 24 h of MV and diaphragm thickening fraction may predict extubation success and prolonged post-extubation non-invasive ventilation [47, 51–53].

## Mitigating the side-effects of mechanical ventilation

**Fig. 1:**
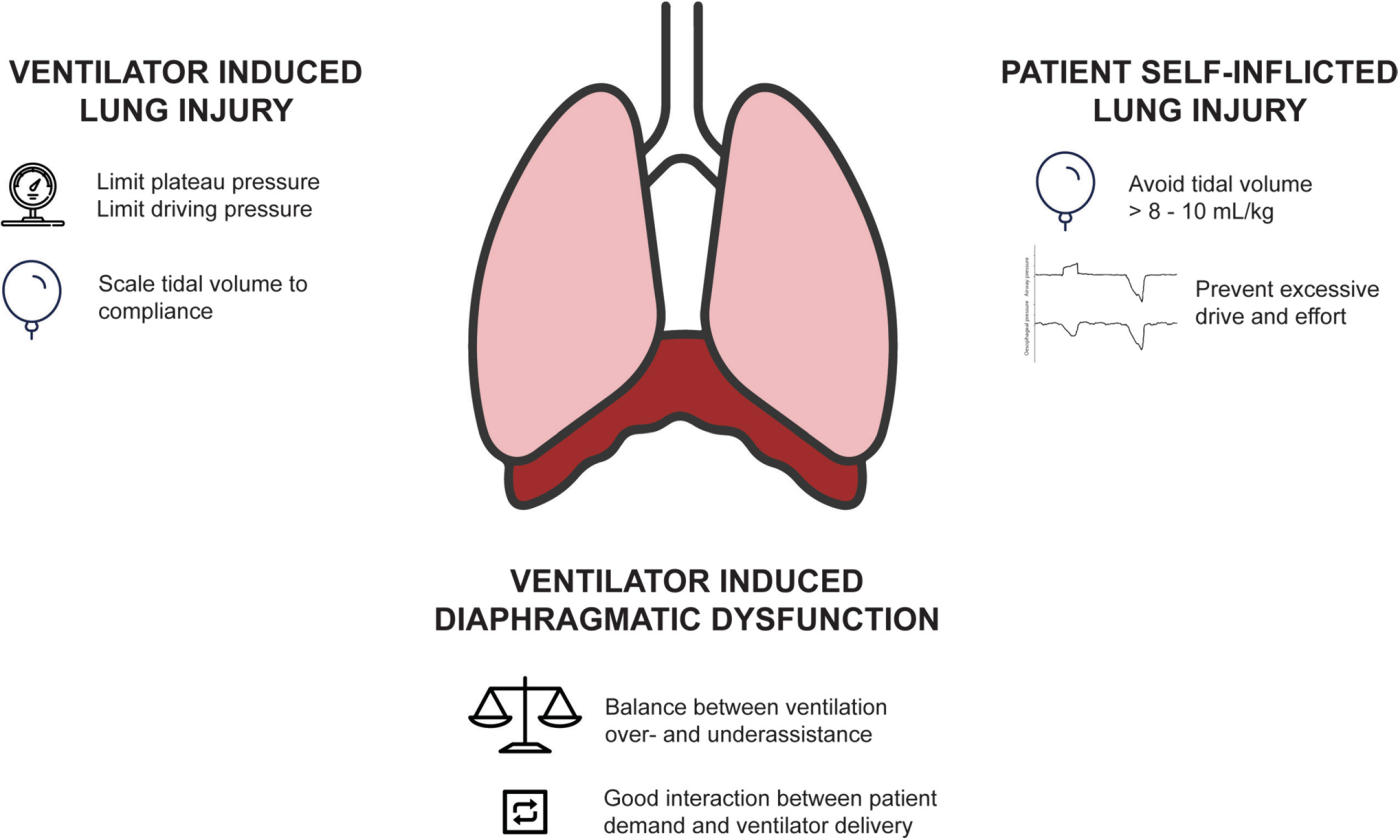
Graphical summary of the main approaches to limiting ventilator-induced lung injury (VILI), ventilator-induced diaphragmatic dysfunction (VIDD), and patient self-inflicted lung injury (P-SILI)

To summarize, VILI, P-SILI, and VIDD have in common that they occur when there is either too much or too little ventilatory assistance. This means that a delicate balance needs to be found in titrating ventilator settings and that this should be done in de context of the phase of the disease trajectory (Fig. 1). Furthermore, VILI, P-SILI, and VIDD cannot be seen as independent from each other as they share some putative mechanisms.

### VILI

Inspiratory pressures must be limited to prevent excessive lung stress. In general, it is recommended to limit plateau pressure (i.e. the airway pressure at zero flow) to 28 cmH_2_O (or 32 cmH_2_O in patients with increased chest wall elastance such as obese patient or patients with stiff chest wall due to oedema). Excessive lung strain can be prevented by either decreasing Vt and/or increasing end-expiratory lung volume (EELV). Physiologic Vt is 5–8 mL/kg, but the actual Vt set should be scaled by respiratory system compliance (Crs). Gattinoni proposed the “baby lung” concept which states that Crs has a linear relationship with the amount of inflatable lung volume. Thus, the lower the Crs, the stiffer the lung and the lower the allowable Vt would be. The importance of scaling Vt to Crs can be appreciated from the landmark Acute Respiratory Distress Syndrome (ARDS) Network trial published in 2000 [13]. In this trial, a low Vt strategy (6 mL/kg predicted bodyweight (PBW)) resulted in a significantly lower mortality compared to a “traditional” Vt strategy (i.e. 12 mL/kg PBW). Post-hoc analysis of individual patient data showed that this mortality benefit was the strongest in subjects with a reduced compliance (i.e. baby lung) at study entry [13, 54]. Pooling adult randomized and controlled Vt trials underscored the assumption that baseline Crs is an important variable in Vt selection [55]. How can at the bedside “best” Vt be selected? Amato et al. reported that increased driving pressure (i.e. the ratio of Vt over Crs) > 15 cmH_2_O was associated with increased mortality risk in adults with ARDS [56]. Decreasing driving pressure by limiting Pplat and increasing PEEP was associated with decreased risk of mortality and that limiting driving pressure was a stronger predictor for outcome than Vt. Therefore, scaling Vt with concurrent targeting driving pressure < 15 cmH_2_O and limiting Pplat < 28 cmH_2_O is the most justified approach [57, 58]. This can most easily be achieved while ventilating the patient in PC mode. In PC ventilation, inspiratory pressures are set so it is easier to reach the target DP; the Vt that will be delivered depends on the Crs and resistance of the respiratory system. The drawback of PC is that there is no inspiratory pause; hence, Pplat is not measured. This requires a manual hold by the operator. Peak inspiratory pressure overestimates Pplat (especially in disease conditions with increased airway resistance) and can therefore not be used to calculate DP unless the inspiratory time is long enough to have zero-flow at end-inspiration [59]. Pressure Regulated Volume Control (PRVC) is a much-cherished mode among paediatric critical practitioner as this most combines the benefits of PC ventilation with volume targeting.

Positive end-expiratory pressure (PEEP) is used to promote more homogenous ventilation by preventing alveolar collapse at end expiration [60]. The potential drawback is that, in combination with the set tidal volume, inappropriately set PEEP may also cause circulatory depression and contribute to VILI through alveolar overdistention during end-inspiration especially when Vt and Pplat and DP limits are not maintained during PEEP selection [58]. The overall effect of PEEP is primarily related to the balance between the number of alveoli that are recruited to participate in ventilation and the amount of lung that is overdistended [61]. The ratio of benefit to harm from PEEP cannot be seen independent from the amount of lung that can be recruited, which varies widely among patients especially those with ARDS [62]. In the abovementioned ARDSNetwork trial, PEEP was titrated according to a table of PEEP and FiO_2_ combinations. In a comparative study, this table proved to be the best approach to PEEP setting in ARDS. In children with more severe lung injury, it was also observed that non-adherence to this table was associated with increased mortality [22, 23, 63]. It is therefore recommended, especially in patients with more severe lung disease, to initially set PEEP according to the grid and then individualize PEEP setting balancing oxygenation and haemodynamics. Lung recruitment manoeuvres (RM) may help to identify which patients might benefit from a higher level of PEEP. The concept of RMs includes an intentional transient increase in transpulmonary pressure aimed at reopening non-aerated or poorly aerated alveoli [64]. There are various types of RM, including sighs, sustained inflation (SI), and a stepwise incremental (± decremental) PEEP titration [65]. With a staircase incremental (± decremental) PEEP titration, with a fixed Vt or DP depending on which ventilation mode is used, PEEP is gradually increased. Evidence for recruitability includes a decrease in Pplat (if in VC mode) or an increase in Vt (in PC mode). Improvements in oxygenation may also be an indicator for lung recruitability with higher PEEP [66].

### P-SILI and VIDD

Preventing P-SILI means that patient respiratory effort needs to be objectively quantified instead of relying on subjective clinical variables. The peak-to-through oesophageal pressure during inspiration is the classic approach for quantifying patient respiratory effort, but this is not readily available, and therefore, non-invasive alternatives such as the occlusion pressure (Pocc) are proposed when oesophageal pressure manometry is unavailable. Pocc can be measured using a simple manoeuvre that is available on many ventilators; the operator performs an expiratory hold while the patient is taking a breath. The peak-to-through change in airway pressure reflects the Pocc and is about 75% of the peak-to-through oesophageal pressure. DP can also be assessed in spontaneously breathing patients, and respiratory drive can be assessed by measuring the drop in airway pressure during the first 100 ms (P0.1) of the Pocc manoeuvre. From a practical perspective, during spontaneous breathing Vt > 8–10 mL/kg PBW should be avoided, especially if coincides with strong inspiratory efforts (i.e. peak-to-through oesophageal pressure > 10–15 cmH_2_O) and respiratory drive (i.e. P0.1 > 4–5 cmH_2_O). Measures to reduce strong inspiratory efforts include switching to a CSV mode of ventilation which allows the patient to take full control, optimisation of sedation, and setting higher PEEP. Aside from this, the patient should be actively examined for extubation readiness through daily spontaneous breathing trials to reduce the duration of MV. From a practical perspective, this entails a daily review of the patient’s ability of maintain sufficient gas exchange without increased work of breathing on minimal support.

## Conclusions

Mechanical ventilation is a double-edged sword. While irrefutable lifesaving for children with acute respiratory failure and a key component in the management of children undergoing elective procedures, when not titrated to the needs of the individual patient MV also bears serious side-effects. Current concepts of the approach to MV for children include limiting lung stress and strain, reducing excessive patient respiratory effort, and finding the delicate balance between ventilatory over- and underassistance to decrease the risk of myotrauma.

## Data Availability

No datasets were generated or analysed during the current study.
